# Annotations

**Published:** 1911-03-18

**Authors:** 


### Annotations.

### The Order of St. John.

In the *London Gazette* appears a list of promotions in the Order of the Hospital of St. John of Jerusalem in England, sanctioned by the King, and dated March 8. Dr. Philip Frank, Major F. A. Brooks, and Lieut.-Col. Edwin Lee are raised from Honorary Associates to be Knights of Grace. These comprise the promotions of medical interest. Of the others there is one new Knight of Justice, nine new Knights of Grace, and two new Esquires. Nearly all these are either Army officers or titled gentlemen. It may be supposed that they have rendered services of some sort to the Order; but it is still true that the work of the Order is mainly carried out by the ungrudging and usually gratuitous services of very many members of the medical profession. That this is the case is indisputable, as is the fact that the higher grades of the Order contain an overwhelming proportion of laymen, most of whom have done but little, as far as the Order is concerned, to deserve those honours. The other orders of chivalry are freely bestowed upon officers of the Army, and quite properly. But that is all the more reason why a better discrimination should be exercised in the Grand Priory of St. John.

### The Fees of Medical Witnesses.

An important case has been heard recently before the Recorder of Dublin. According to the *Journal of the Irish Medical Asociation*, it was seriously argued by the defence that once a doctor had examined a patient whose case is or may be the subject of litigation in the courts, he (the doctor) is liable to be summoned as a witness and can be compelled to attend and give evidence in court on payment to him of one guinea. Whether this is in fact the law was not actually decided by the Recorder, for the plaintiff in the case, Sir Christopher Nixon, Bart., in accepting the case and undertaking to give evidence had definitely fixed his fee beforehand, and thus bound the defendant by a contract. He attended the court at the time fixed to give evidence, but the case was settled between the parties and his evidence was not required. The Recorder gave judgment for the full amount claimed, deciding that this circumstance in no way affected the physician's rights. This decision is of importance, in that if the contention made by the defence had proved of any weight in the mind of the judge medical men might find themselves subjected to very unwelcome experiences. It is easy to see that one of the first results of the establishment of the doctrine held by the defence would be an extreme unwillingness on the part of the doctor to attend the patient in the first instance.

### The Treatment of Medical Witnesses.

Lower human nature has a good many little ways of getting even (or of persuading itself it gets even) with its betters. The recent happily slain morganatic dragon forms an instance known to everyone. But truly to every highly-placed person are attached slanderous rumours, as that one statesman or general drinks, and another does something worse—and very particularly that the credit of this or that distinguished man's achievement belongs, if everyone had their rights, to some unfortunate underling. Of this last form of calumny we of the medical household are not guiltless, and never have been and never will be. After this admission, it may perhaps be pointed out that something very like the foregoing is at the bottom of the obvious dislike shown to the expert witness in the Law Courts. A sense of inferiority in knowledge of the subject is bound to put some of his interlocutors on the offensive, especially as legal advocates are combatively disposed, and, moreover, accustomed to think that a very fair opinion in any branch of human knowledge can be passed after an hour or two with a popular compendium of the subject in question. The medical expert comes off no better than the others. It may be remembered that the judge in the Crippen trial told a high medical authority, who had given his evidence in a businesslike way, with no tendency at all to prolixity, that in the matter of a further statement asked for the court did not want a medical lecture. In the recent action against a bonesetter, Sir Edward Carson's inexpensive jests about the tubercle bacillus are in just the same taste. No doubt a law court is often a tedious place enough, but it should be possible to get diversion somehow without sacrificing courtesy. The days of Buzfuz are past, and the relative positions of a scientific and of a non-scientific profession beginning to change.

### The Medical Navy League.

Every well-read medical man must have formed a great respect for the enterprise, industry, and thoroughness characteristic of certain Continental nations; and further, from frequent bitter experience of being summoned out of it, he must wish at least as much as anyone to sleep soundly in his bed. But, in spite of these admitted facts, where is the fitness in the medical profession interesting itself directly in matters of public policy which have not the slightest bearing upon medicine—as one or two metropolitan consultants have shown themselves actively desirous of its doing? There are, indeed, plenty of affairs of State in which medical men should, and in the countries referred to do, have a much larger say. Here it is common for bishops or politicians to take the chair and make the speeches at gatherings which should be directed by the leaders of our profession. But two blacks do not make a white, and no good will result from trying in our turn to mind other people's business. We see that all implication of politics is disclaimed, but such an association must inevitably have a political flavour. This is, however, not the principle involved, which simply consists in the fact that the galley of naval propagandism is no place for the medical man as such.

